# The Medical Argus

**Published:** 1891-01

**Authors:** 


THE MEDICAL ARGUS. A monthly journal, published by Dr. F. F. Casseday, 211 and 212 Keith & Perry Building, Kansas City, Missouri.

This is a new journal devoted to Homœopathy. The number before us is the fifth issue. It contains several interesting articles—one on Medical Jurisprudence, by Dr. L. E. Russell; one on Protection of the Public against Tubercular Consumption, by Dr. Pemberton Dudley; and a letter, one of a series, by Dr. Charles N. Hart, upon How to Visit the Medical Attractions of London.

